# Comparison of treatment methods for submacular hemorrhage in neovascular age-related macular degeneration: conservative versus active surgical strategy

**DOI:** 10.1038/s41598-022-18619-5

**Published:** 2022-09-01

**Authors:** Yongseok Mun, Kyu Hyung Park, Sang Jun Park, Han Joo Cho, Chul Gu Kim, Jong Woo Kim, Dong Geun Park, Min Sagong, Jae Hui Kim, Se Joon Woo

**Affiliations:** 1 Department of Ophthalmology, Hallym University College of Medicine, Kangnam Sacred Heart Hospital, 665-3, Siheung-daero, Yeongdeungpo-gu, Seoul, 07442 South Korea; 2 Department of Ophthalmology, Seoul National University College of Medicine, Seoul National University Bundang Hospital, 82, Gumi-ro 173 Beon-gil, Bundang-gu, Seongnam-si, Gyeonggi-do 13620 South Korea; 3 Department of Ophthalmology, Kim’s Eye Hospital, 136, Yeongshin-ro, Yeongdeungpo-gu, Seoul, 07301 South Korea; 4 Department of Ophthalmology, Yeungnam University College of Medicine, 170, Hyunchung-ro, Nam-gu, Daegu, 42415 South Korea

**Keywords:** Macular degeneration, Outcomes research

## Abstract

The optimal treatment of submacular hemorrhage (SMH) following neovascular age-related macular degeneration (nAMD) is controversial. This study aimed to compare visual outcomes of conservative versus active surgical treatment. Two hundred thirty-six eyes of 236 patients with SMH (≥ 1 disc diameter) were stratified into four groups: observation (n = 21); anti-vascular endothelial growth factor (VEGF) monotherapy (n = 161); non-surgical gas tamponade (n = 31); and subretinal surgery (n = 23). The primary outcome was best-corrected visual acuity (BCVA) at 12 months. The baseline BCVAs of the observation, anti-VEGF monotherapy, non-surgical gas tamponade, and subretinal surgery groups were 1.50 ± 0.70, 1.09 ± 0.70, 1.31 ± 0.83, and 1.62 ± 0.77 logarithm of minimal angle resolution (LogMAR), respectively. The mean BCVAs at 12 months were 1.39 ± 0.84, 0.90 ± 0.83, 1.35 ± 0.88, and 1.44 ± 0.91 LogMAR, respectively. After adjusting for age, baseline BCVA, SMH size, and the number of intravitreal anti-VEGF injections before SMH, the mean BCVA showed no significant difference among treatments at 12 months (*P* = 0.204). The anti-VEGF monotherapy group showed better mean BCVA significantly at 3 months (*P* < 0.001). Only baseline BCVA was associated with VA gain at 12 months (Odds ratio = 3.53, *P* < 0.001). This study demonstrated that there was no difference in 12 month visual outcomes among treatments and a better early visual outcome can be expected with anti-VEGF monotherapy.

## Introduction

Submacular hemorrhage (SMH) in neovascular age-related macular degeneration (nAMD) is a sight-threatening complication that has a poor visual prognosis. It results in  damages to the retina and retinal pigment epithelium (RPE) cells^1,2^. Subsequent subretinal fibrosis and macular atrophy cause irreversible vision loss^3,4^. An average of three or more lines of vision decrease as the natural course of untreated patients^5^. Scupola et al. found that 90% of eyes with SMH secondary to nAMD had a VA worse than 20/200^3^.

For treating SMH secondary to AMD, many non-surgical and surgical methodologies have been proposed, including observation, intravitreal anti-vascular endothelial growth factor (VEGF) injection, pneumatic displacement, subretinal or intravitreal tissue plasminogen activator (t-PA) injection, and photodynamic therapy^6–12^. Anti-VEGF can help to clear SMH and reduce the risk of recurrence^13,14^. The pneumatic displacement of SMH is expected to reduce exposure of iron toxicity and RPE detachment from the retina, which leads to outer retinal damage^6,10^. Intravitreal t-PA or subretinal t-PA may help liquefy the clot and aid pneumatic displacement. Either vitrectomy can evacuate SMH or t-PA can be injected subretinally to displace SMH using expansile gas^15,16^.

However, controversies still exist regarding an optimal therapeutic option to treat SMH associated with nAMD. Most studies reported an interventional case series with the effectiveness of one methodology or a comparison of two non-surgical methodologies. A few studies with small number of patients compared surgical treatment with non-surgical treatment^15,16^. However, most previous studies were conducted with one or two treatment methods rather than three or more^6–11,13–20^.

Our investigation aimed to compare visual outcomes in three non-surgical strategies and one surgical (active) treatment strategy—observation, intravitreal anti-VEGF injection, non-surgical pneumatic displacement, and vitrectomy with subretinal t-PA and pneumatic displacement.

## Methods

This was a retrospective, multicenter, interventional study in which two tertiary referral hospitals, Seoul National University Bundang Hospital (SNUBH), Yeungnam University Medical Center (YUMC), and one specialized eye center, Kim’s Eye Hospital (KEH), participated. The study protocol was approved by all institutional review boards (IRBs) and informed consent was waived because of its retrospective nature (Seoul National University Bundang Hospital IRB: No. B-1905-543-110, Yeungnam University Medical Center IRB: No. 2019-06-041, Kim’s Eye Hospital IRB: No. 2019-07-012). The study adhered to the tenets of the Declaration of Helsinki.

We conducted a retrospective review of the medical records of patients who met all of the following inclusion criteria between January 2006 and December 2019: (1) significant SMH secondary to nAMD involving the fovea with a size equal to or greater than 1 disc diameter (DD); (2) therapeutic details can be obtained; and (3) follow-up for at least 3 months. Cases of SMH secondary to other retinal disease, such as retinal macroaneurysm, myopia or other retinal diseases were excluded.

All subjects underwent comprehensive ophthalmologic examinations, including measurement of best-corrected visual acuity (BCVA), slit-lamp biomicroscopy, fundus photography, and optical coherence tomography (Spectralis OCT, Heidelberg Engineering) at every visit. Diagnoses of nAMD were based on fundus examination, OCT, fundus fluorescein angiography, and indocyanine green angiography (HRA-2, Heidelberg Engineering, Heidelberg, Germany). The size of the SMH was also evaluated using conventional and ultra-wide field fundus photography for the approximate number of disc diameters at the initial visit. The number of intravitreal anti-VEGF injections before SMH and the time-to-treatment (the time from diagnosis of SMH to initiating treatment) were also collected. Central foveal thickness data were obtained, but they were not used for analysis because in many cases, baseline values could not be measured due to thick SMH in many cases. In many cases, it was difficult to distinguish between typical nAMD and polypoidal choroidal vasculopathy (PCV) since the SMH and subretinal scarring disturbed interpretation of angiography.

The initial treatment strategy was determined according to the physician’s discretion and all patients were treated consecutively at each institution. Eyes were only followed without treatment, *observation group*; eyes were administered intravitreal injection of anti-VEGF agents, 1.25 mg bevacizumab, 0.5 mg ranibizumab, or 2 mg aflibercept, *anti-VEGF monotherapy group*; eyes were administered intravitreal pure gas injection (either 0.5 mL SF_6_ or 0.25 mL C_3_F_8_) combined with or without intravitreal anti-VEGF or t-PA, *non-surgical gas tamponade group*. Patients were instructed to maintain the prone position for at least 3 days; and eyes which underwent vitrectomy, subretinal t-PA injection and pneumatic displacement combined with or without intravitreal anti-VEGF injection, *subretinal surgery group*. Patients who were not expected to regain their visual functions due to massive SMH (its size was over 7 DD and its thickness could not be measured in OCT) were only followed up without any treatment and categorized as observation group. Patients with vision-threatening breakthrough vitreous hemorrhage or retinal detachment underwent rescue vitrectomy regardless of the treatment strategy. After initial treatment, anti-VEGF was injected as required (pro re nata) with at least 1-month interval in all groups.

The primary outcomes were the mean BCVA from baseline at 12 months. Secondary outcomes were mean VAs at 3 and 6 months after the initial treatment. Snellen BCVAs were converted to logarithm of minimum angle resolution (LogMAR) values. Counting fingers and hand motion were considered to indicate visual acuities of 1.85 LogMAR and 2.28 LogMAR, respectively for statistical analyses^21^. We performed subgroup analyses according to baseline BCVAs since a previous study reported that it affected final visual acuity^22^. We conducted subgroup analysis according to the baseline VA: (1) poor: ≥ 2.0 LogMAR; (2) intermediate: < 2.0 and ≥ 1.0 LogMAR; (3) good: LogMAR, < 1.0 LogMAR. We defined VA gain and loss of ≥ 3 lines (0.3 LogMAR) from baseline BCVA. Another subgroup analysis according to the size of SMH was also conducted: (1) SMH size ≤ 4 DD; (2) SMH size > 4 DD.

Statistical analyses were performed using SPSS software for Windows (version 26.0; SPSS Inc., Chicago, IL). Differences in visual acuities among treatment strategies were analyzed using the ranked (non-parametric) analysis of covariance (ANCOVA). Continuous variables were analyzed using analysis of variance or Kruskal–Wallis test. Categorical values were analyzed using the chi-square test. Statistical significance was set at *P* < 0.05.

## Results

Two hundred and thirty-six patients (236 eyes) who developed SMH due to nAMD were enrolled in this study. There were 21, 161, 31, and 23 patients in the observation group, anti-VEGF monotherapy group, non-surgical gas tamponade group, and subretinal surgery group, respectively. Table 1 shows the differences in the demographic and clinical characteristics among the treatment strategies.

**Table 1:** Demographic and clinical characteristics for submacular hemorrhage patients with neovascular age-related macular degeneration.

	Observation (n = 21)	Anti-VEGF monotherapy (n = 161)	Non-surgical gas tamponade (n = 31)	Subretinal surgery (n = 23)	P value
Age, mean (SD)	66.73 (10.69)	70.17 (10.39)	69.74 (11.32)	75.96 (9.25)	0.026*
**Sex, no. (%)**					
Male	17 (81)	102 (63)	22 (71)	14 (61)	0.512^†^
Female	4 (9)	59 (37)	9 (29)	9 (39)	
**Comorbidity, no. (%)**					
Diabetes mellitus	4 (19)	32 (20)	6 (19)	1 (4)	0.332^‡^
Hypertension	13 (62)	76 (47)	13 (42)	13 (57)	0.527^†^
Cerebrovascular disease	1 (5)	8 (5)	0 (0)	0 (0.0)	0.558^‡^
Heart disease	2 (10)	17 (11)	1 (3)	0 (0.0)	0.513^‡^
Kidney disease	0 (0)	3 (2)	0 (0)	0 (0.0)	1.000^‡^
Cancer	2 (10)	8 (5)	1 (3)	0 (0.0)	0.562^‡^
Others	2 (10)	22 (14)	4 (13)	0 (0.0)	0.279^‡^
Baseline LogMAR BCVA, mean (SD)	1.57 (0.74)	1.09 (0.70)	1.31 (0.83)	1.62 (0.77)	0.001*
Size of SMH, Disc diameter, mean (SD)	11.14 (2.63)	7.92 (6.01)	5.86 (4.60)	4.84 (2.04)	< 0.001**
The number of intravitreal anti-VEGF before SMH	0.29 (1.10)	0.19 (0.88)	0.48 (1.91)	2.70 (6.07)	0.001**
Time-to-treatment, days, mean (SD)	–	8.56 (17.7)	7.45 (8.76)	11.1 (19.0)	0.722
Rescue vitrectomy, no. (%)	8 (38)	11 (7)	8 (26)	1 (4)	** < 0.001**^‡^

VEGF, vascular endothelial growth factor; t-PA, tissue plasminogen activator; SD, standard deviation; LogMAR, logarithm of the minimum angle of resolution; BCVA, best-corrected visual acuity; SMH, submacular hemorrage.

*t-test.

^†^chi-square test.

^‡^Fisher’s exact test.

**Kruskal–Wallis test.

P values in bold are statistically significant.

Patients in the observation group were younger than those in the other groups (*P* = 0.026). The intravitreal anti-VEGF group showed better baseline BCVA and the subretinal surgery group showed worse baseline BCVA than the other groups (*P* = 0.001). The size of SMH was larger in the observation group and smaller in the subretinal surgery group than the other groups (*P* < 0.001). Before the occurrence of SMH, anti-VEGF was injected more frequently in the subretinal surgery group. (*P* < 0.001). These four variables were considered as covariates for the analysis of VA using ranked ANCOVA. Rescue vitrectomies were performed in 36.4% of observation, 6.8% of anti-VEGF monotherapy, 25.8% of non-surgical gas tamponade, and 4.3% of the subretinal surgery group.

**Figure 1:**
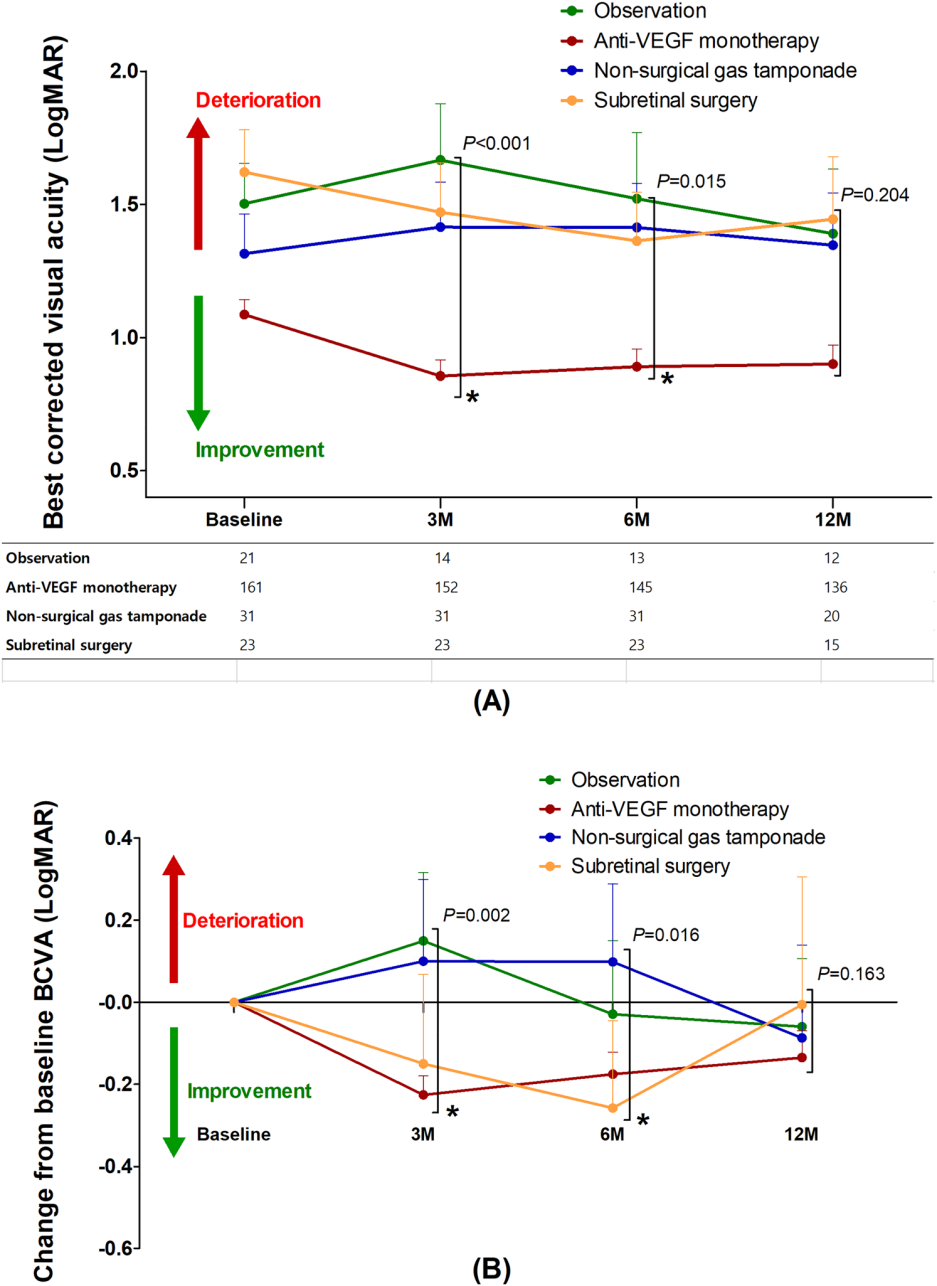
The visual outcomes in eyes treated for submacular hemorrhage secondary to neovascular age-related macular degeneration according to each treatment strategy. (**A**) The trend of best-corrected visual acuity (BCVA). (**B**) The trend of BCVA change from baseline. LogMAR, logarithm of the minimum angle of resolution; M, months.

Figure 1A represents the mean BCVAs at baseline and their changes at 3, 6 and 12 months between the treatment groups. The mean BCVAs of observation, anti-VEGF monotherapy, non-surgical gas tamponade and subretinal surgery group at 12 months were 1.39 ± 0.84, 0.90 ± 0.83, 1.35 ± 0.88, and 1.44 ± 0.91, respectively (see Supplementary Table S1). After adjusting for four covariates, there was no significant difference among the treatment strategies (*P* = 0.204) at 12 months. The mean BCVAs among groups were significantly different at 3 and 6 months, and the anti-VEGF monotherapy group showed better VA at 3 and 6 months and greater BCVA gain than the other groups at 3 months (Fig. 1B).

### Subgroup analysis

**Figure 2:**
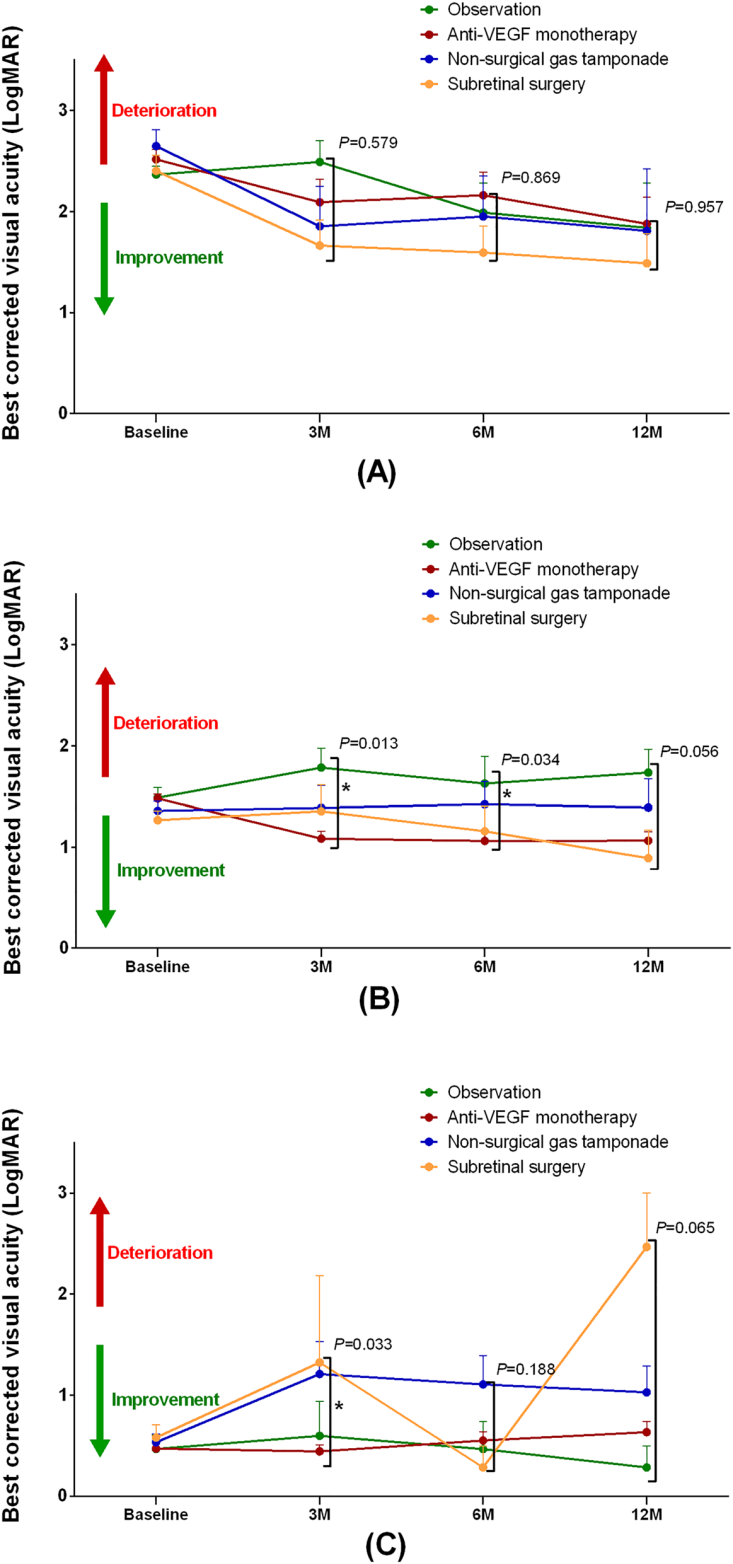
The subgroup anaylsis based on baseline best-corrected visual acuities (BCVA). (**A**) Poor: Baseline BCVA ≥ 2.0 LogMAR (Baseline BCVA ≤ 20/400). (**B**) Intermediate: 1.0 LogMAR ≤ Baseline BCVA < 2.0 LogMAR (20/400 < Baseline BCVA ≤ 20/200). (**C**) Good: Baseline BCVA < 1.0 LogMAR (Baseline BCVA > 20/200). LogMAR, logarithm of the minimum angle of resolution; M, months.

In the poor baseline BCVA group, the mean BCVAs at 12 months of observation, anti-VEGF monotherapy, non-surgical gas tamponade, and subretinal surgery groups were 1.84 ± 0.62, 1.88 ± 0.70, 1.81 ± 1.23 and 1.49 ± 0.72, respectively (see Supplementary Table S1). After adjustment of covariates, there was no significant difference among groups in mean BCVA at 3, 6 and 12 months (P = 0.579, 0.869 and 0.957, respectively) (Fig. 2A). In the intermediate baseline BCVA group, the mean BCVAs at 12 months of observation, anti-VEGF monotherapy, non-surgical gas tamponade, and subretinal surgery group were 1.73 ± 0.60, 1.06 ± 0.71, 1.39 ± 0.85 and 0.89 ± 0.68, respectively. After covariate adjustment, a significant difference in mean BCVA among groups was observed at 3 and 6 months, and the anti-VEGF monotherapy group showed better BCVA (*P* = 0.013 and 0.034, respectively) (Fig. 2B). In the good baseline BCVA group, the mean BCVAs at 12 months of observation, anti-VEGF monotherapy, non-surgical gas tamponade, and subretinal surgery groups were 0.28 ± 0.37, 0.63 ± 0.83, 1.03 ± 0.69 and 2.47 ± 0.92, respectively. After covariate adjustment, a significant difference in mean BCVA among the groups was observed at 3 months (*P* = 0.033) (Fig. 2C). In particular, the mean BCVA of the subretinal surgery group was worse than that of the other three groups at 12 months.

**Figure 3:**
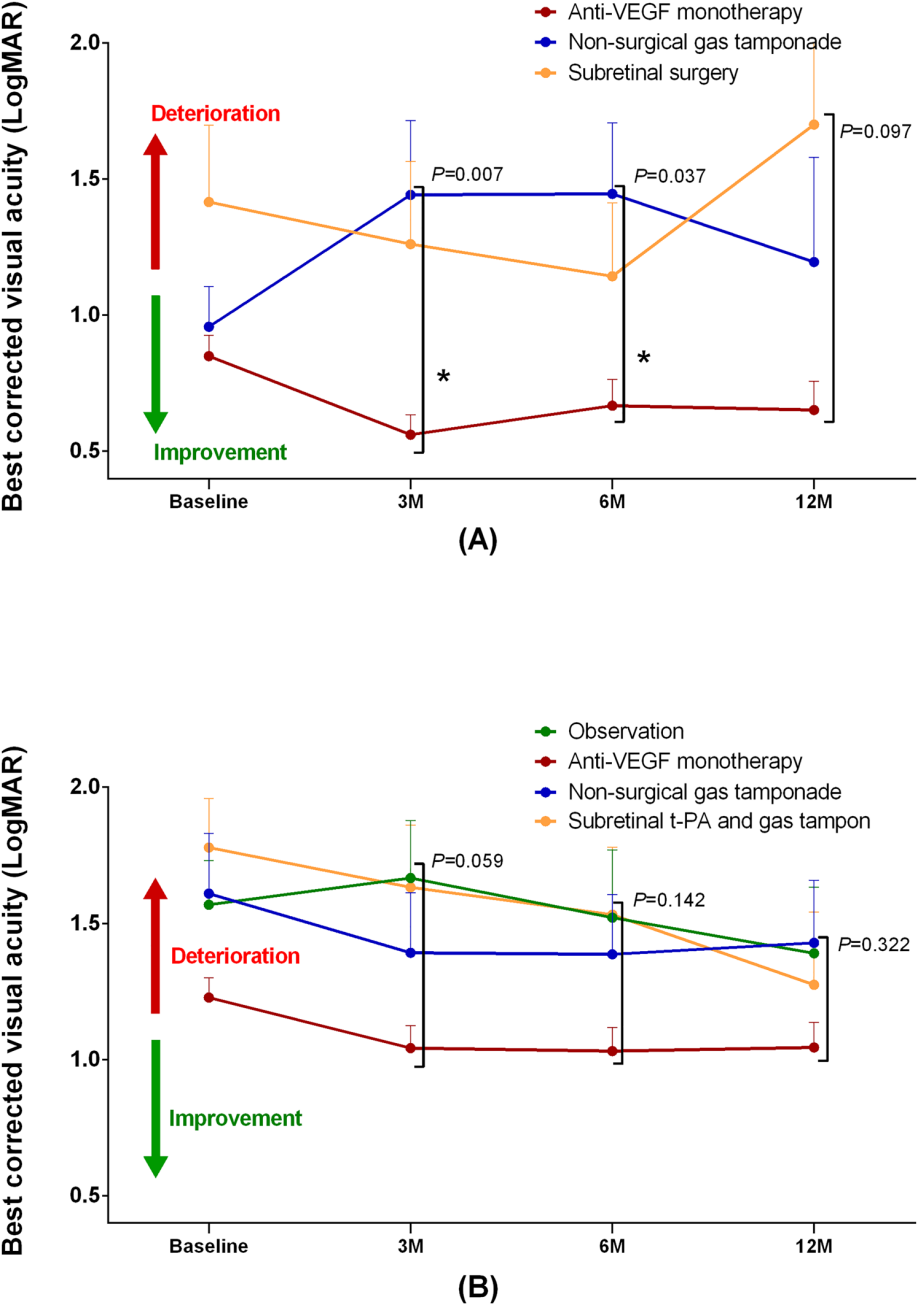
The subgroup anaylsis by the size of submacular hemorrhage. (**A**) Size of submacular hemorrhage ≤ 4 disc diameter. (**B**) Size of submacular hemorrhage > 4 disc diameter. LogMAR, logarithm of the minimum angle of resolution; M, months.

In patients with SMH size ≤ 4 DD, the mean BVCA at 12 months of anti-VEGF monotherapy, non-surgical gas tamponade, and subretinal surgery groups were 0.65 ± 0.74, 1.20 ± 1.01, and 1.70 ± 1.07, respectively (see Supplementary Table S1). There was no patient with SMH size ≤ 4 DD in the observation group. After covariate adjustment, we found significant differences among groups in BCVAs at 3 and 6 months (*P* = 0.007 and 0.037, respectively) and the anti-VEGF monotherapy group had significantly better BCVAs at 3 and 6 months than the other groups. The anti-VEGF monotherapy group showed visual improvement at 3, 6, and 12 months compared to the baseline while the other groups showed visual deterioration (Fig. 3A) (see Supplementary Table S1). The mean BCVAs deteriorated in the non-surgical gas tamponade and subretinal surgery groups. In patients with SMH size > 4 DD, the mean BCVAs at 12 months of observation, anti-VEGF monotherapy, non-surgical gas tamponade, and subretinal surgery groups were 1.39 ± 0.84, 1.04 ± 0.84, 1.43 ± 0.83, and 1.27 ± 0.80, respectively. No significant differences were found among groups in the mean BCVAs at 3, 6, and 12 months (*P* = 0.059, 0.142, and 0.322, respectively) (Fig. 3B).

### Gain and loss in visual acuities

**Figure 4:**
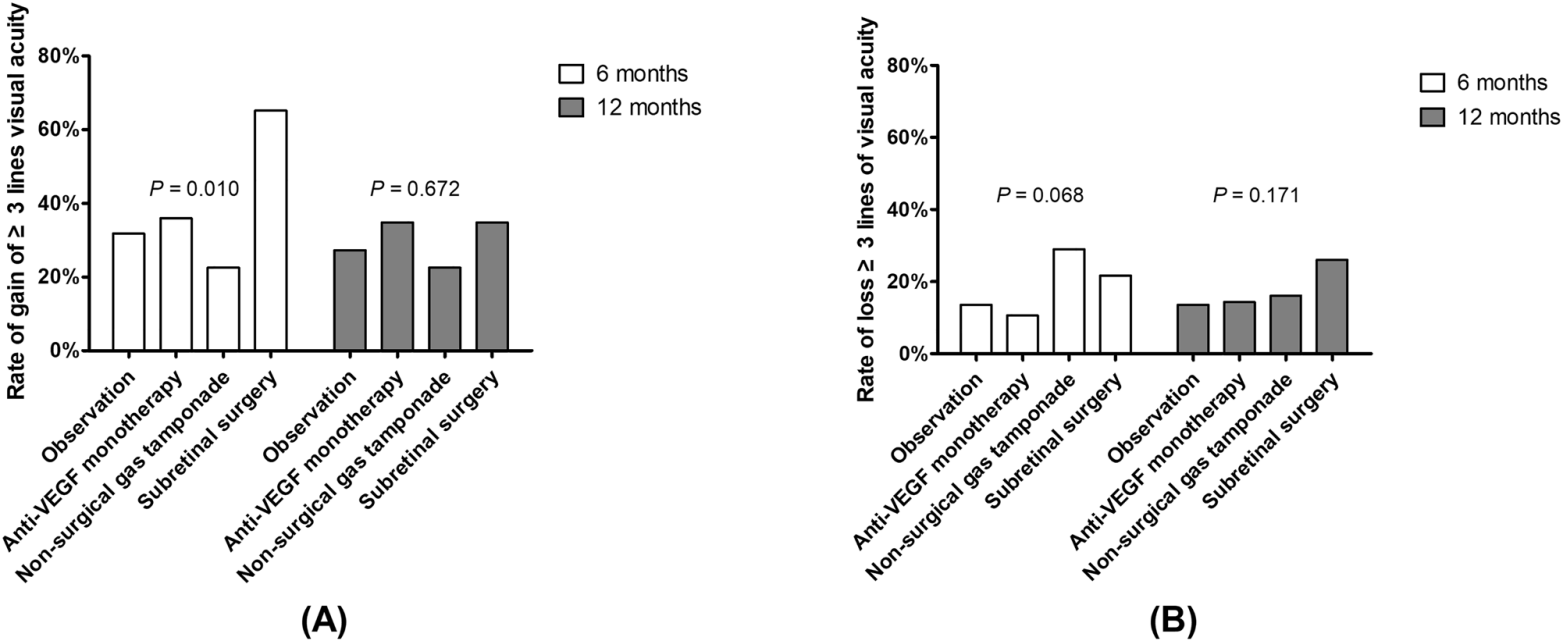
Rate of loss or gain of visual acuity. (**A**) Gain of ≥ 3 lines (**B**) Loss of ≥ 3 lines. VEGF, vascular endothelial growth factor.

**Figure 5:**
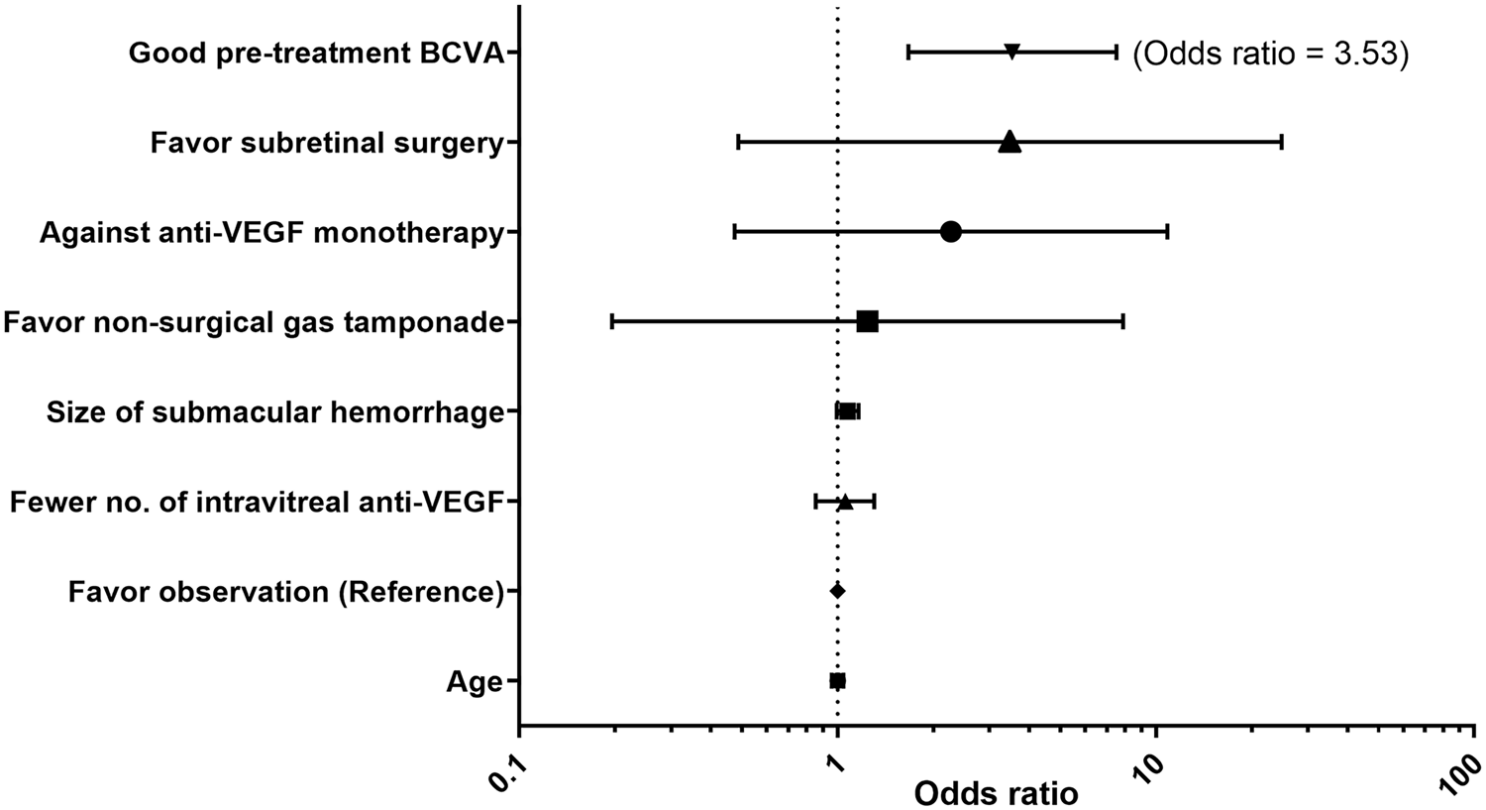
Logistic regression model for gain of ≥ 3 lines in visual acuity at 12 months after treatment. VEGF, vascular endothelial growth factor; BCVA, best-corrected visual acuity.

Figure 4 illustrate the rate of VA gain and loss of ≥ 3 lines. There was no significant difference in the rate of VA gain at 12 months (*P* = 0.672) (Table 2 and Fig. 4). However, a significant difference in VA gain was observed at 6 months, and the subretinal surgery group showed a higher rate of VA gain than the other groups (65.2%) (*P* = 0.010). Non-surgical gas tamponade (29.0%) and subretinal surgery group (26.1%) showed higher rates of VA loss at 6 and 12 months, respectively, but there was no significant difference among groups at 6 and 12 months (*P* = 0.068 and 0.171, respectively). In multivariate logistic regression analysis, only baseline BCVA was associated with VA gain 12 months after the initial treatment (*P* < 0.001) (Fig. 5).

**Table 2:** Rates of loss or gain of visual acuity at 6 and 12 months after treatment for submacular hemorrhage in neovascular age-related macular degeneration.

Visual acuity	Observation (n = 21)	Anti-VEGF monotherapy (n = 161)	Intravitreal gas tamponade (n = 31)	Subretinal surgery (n = 23)	*P* value*
**Gain of ≥ 3 lines**^**†**^					
After 6 months, No. (%)	7 (33)	58 (36)	7 (23)	15 (65)	0.010
After 12 months, no. (%)	6 (29)	56 (35)	7 (23)	8 (35)	0.672
**Loss of ≥ 3 lines**					
After 6 months, No. (%)	3 (14)	17 (11)	9 (29)	5 (22)	0.068
After 12 months, no. (%)	3 (14)	23 (14)	5 (16)	6 (26)	0.171

VEGF, vascular endothelial growth factor; t-PA, tissue plasminogen activator; LogMAR, logarithm of the minimum angle of resolution.

*Chi-square test.

^†^3 lines = 0.3 LogMAR = EDTRS 15 letters.

## Discussion

This study compared the visual acuities at 1-year after initial treatment according to four treatment strategies: observation, anti-VEGF monotherapy, non-surgical gas tamponade, and subretinal surgery group. We found no significant difference in the mean BCVA at 12 months after the initial treatment. Significant differences were found at 3 and 6 months and the anti-VEGF monotherapy group showed significant and the greatest improvement. The visual outcomes of four treatment strategies differed according to the baseline BCVA and the SMH size. In the good or intermediate baseline BCVA group, the anti-VEGF monotherapy group showed significant and the greatest improvement at 3 and/or 6 months. In the good baseline BCVA group, the subretinal surgery group showed a marked deterioration in BCVA. In eyes with small baseline SMH, anti-VEGF monotherapy showed better visual outcome at 3 and 6 months, while there was no difference in eyes with large baseline SMH.

Most clinical trials for nAMD did not include patients with significant baseline SMH; thus, there is no treatment guideline for nAMD with baseline SMH. Most previous studies of SMH treatment reported visual outcomes with one treatment strategy, and a few studies compared two of them with a small number of subjects (Table 3). Besides, no previous studies included a comparison group of no treatment.

**Table 3:** Previous studies of various treatment modalities for submacular hemorrhange in neovascular age-related macular degeneration.

Study	Number of subjects	Study design	Treatment strategies	Baseline LogMAR, BCVA, mean (SD)	LogMAR Visual Outcome, mean (SD), follow-up period	Result
Kim et al., 2014	91	Retrospective, interventional	Intravitreal anti-VEGF	1.38 (0.53)	0.96 (0.65), 6 months	Beneficial
Iacono et al., 2014	23	Prospective, interventional	Intravitreal anti-VEGF	0.82 (0.22)	0.68 (0.41), 12 months	Beneficial
Shin et al., 2015	82	Retrospective, comparative, interventional	(1) Intravitreal anti-VEGF	1.21 (0.71)	0.76 (0.59), 6 months	Both are beneficial. Combination therapy was better in thick submacular hemorrhage.
			(2) Intravitreal anti-VEGF with pneumatic displacement	1.14 (0.63)	0.94 (0.61), 6 months	
Cho et al., 2015	93	Retrospective, comparative, interventional	(1) Intravitreal anti-VEGF	1.18 (0.57)	0.96 (0.39), 12 months	Both are beneficial. No difference between them
			(2) Intravitreal anti-VEGF with pneumatic displacement	1.20 (0.74)	0.95 (0.48), 12 months	
Kitagawa et al., 2016	20	Prospective, interventional	Intravitreal t-PA, anti-VEGF, and gas tamponade	0.66 (0.40)	0.42 (0.45), 6 months	Beneficial
Kadonosono et al., 2015	13	Prospective, interventional	Subretinal surgery with t-PA and gas tamponade	1.21 (0.44)	0.70 (0.33), 3 months	Beneficial
Chang et al., 2014	101	Retrospective, comparative, interventional	(1) Subretinal surgery with t-PA, gas tamponade, and intravitreal anti-VEGF	1.92 (N/A)	1.74 (N/A), 6 months	Both are beneficial. Anti-VEGF may help maintain the visual acuity gains.
			(2) Subretinal surgery with t-PA, gas tamponade	2.26 (N/A)	1.54 (N/A), 6 months	
Jong et al., 2016	24	Prospective, comparative, interventional	(1) Intravitreal t-PA, anti-VEGF, and gas tamponade	0.91 (N/A)	0.63 (N/A), 12 weeks	Both are beneficial. No difference between them
			(2) Subretinal surgery with t-PA, gas tamponade, and intravitreal anti-VEGF	0.75 (N/A)	0.72 (N/A), 12 weeks	
Current study	236	Retrospective, comparative, interventional	(1) Observation	1.57 (0.74)	1.39 (0.84), 12 months	No differences among 4 treatment strageis. Intravitreal Anti-VEGF helped with visual recovery in the short term.
			(2) Intravitreal anti-VEGF	1.09 (0.70)	0.90 (0.83), 12 months	
			(3) Intravitrea gas tamponade(± anti-VEGF or t-PA)	1.31 (0.83)	1.35 (0.88), 12 months	
			(4) Subretinal surgery with t-PA, gas tamponade (± anti-VEGF)	1.62 (0.77)	1.44 (0.91), 12 months	

LogMAR, logarithm of minimum angle resolution; SD, standard deviation; VEGF, vascular endothelial growth factor; t-PA, tissue plasminogen activator.

For anti-VEGF monotherapy, Kim et al. (n = 91) revealed that the mean VA improved at 6 months significantly^9^. The mean VA improved from 1.38 ± 0.53 to 0.96 ± 0.65 logMAR, with 59.3% of cases showing gain of ≥ 3 lines in VA. Superiority of anti-VEGF therapy as compared to subretinal surgery or photodynamic therapy, has been previously reported^7,9,23^. Iacono et al. (n = 23) also reported significant improvement of VA (from 0.82 ± 0.22 to 0.68 ± 0.41 logMAR) with ranibizumab alone at 12 months^17^.

For non-surgical pneumatic displacement, Shin et al. (n = 82) reported that anti-VEGF with pneumatic displacement rapidly improved VA (from 1.14 ± 0.63 to 0.94 ± 0.61 logMAR) in eyes with SMH but final VAs were not different between groups with and without pneumatic displacement at 6 months after treatment^10^. Cho et al. (n = 93) also revealed that no significant difference was observed in VA between ranibizumab monotherapy and a combination of ranibizumab and pneumatic displacement at 12 months^6^. Kitagawa et al. (n = 20) found that recombinant t-PA, ranibizumab, and pneumatic displacement without vitrectomy was an effective treatment for SMH and the mean VA gain was 13 ETDRS letters (nearly 0.26 logMAR) at 6 months after treatment^18^.

For vitrectomy with subretinal t-PA and pneumatic displacement, Kadonosono et al. (n = 13) reported VA gain of 23.3 letters at 3 months after treatment^19^. Chang et al. (n = 101) revealed a mean VA change from 2.05 logMAR to 1.76 logMAR at 12 months after treatment^8^. This study also suggested that post-operative anti-VEGF injection might help maintain VA gains. Jong et al. (n = 24) reported that there was no difference between eyes treated with subretinal and intravitreal recombinant t-PA combined with bevacizumab and pneumatic displacement^16^. Those multiple therapeutic approaches resulted in successful but variable visual outcomes. However, an evidence for optimal options or their combinations is still lacking.

Several studies have compared the visual outcomes between two different treatment methods for nAMD-associated SMH. Cho et al. and Shin et al. reported that no significant difference was observed in VA improvement between anti-VEGF monotherapy and non-surgical pneumatic displacement at 6 and 12 months^6,10^. Jong et al. also showed that there was no difference in VA improvement between non-surgical pneumatic displacement and subretinal surgery at 12 weeks^16^. Those results are in line with our study that there was no difference among four treatment strategies in 1-year VA. Prospective studies with a randomized, parallel assignment of patients to compare treatment strategies are now ongoing and are expected to reveal further perspectives (https://clinicaltrials.gov, NCT04663750, NCT01835067, and NCT02557451).

It is well known that SMH results in separating retina from the RPE and it may block the exchange of nutrients and metabolites and induce retinal damage^1^. Toxic effect from iron also accelerates the destruction of photoreceptors^1^. Based on those findings, more aggressive treatment including pneumatic displacement with or without t-PA seems to be helpful. However, we could not find the advantage of pneumatic displacement or subretinal t-PA in our study. It may result from time-to-treatment. Retinal degeneration above SMH occurs at approximately 3 to 14 days in an experimental model^2^. In this study, the average time-to-treatment was 8.56 ± 17.7, 7.45 ± 8.76, and 11.1 ± 19.0 days for anti-VEGF monotherapy, non-surgical pneumatic displacement, and subretinal surgery group, respectively. Therefore, irreversible retinal degeneration or photoreceptor destruction must have progressed beyond a certain level in most cases and it was thought to be a much more significant factor than the treatment strategy. Furthermore, subretinal t-PA can lyse subretinal blood clots enough to be displaced, but it cannot reduce SMH completely^16^. Irreversible RPE defects can lead to atrophy of the subfoveal choriocapillaris and poor visual outcome^20^. The toxicity of t-PA to the outer retina and RPE also has a negative effect on improving visual outcome, and the subretinal route is the most vulnerable way due to its direct exposure^24,25^. Cataract progression after vitrectomy can also limit visual improvement in phakic eyes. The relatively high incidence of hemorrhagic postoperative complications in subretinal surgery, such as hyphema and vitreous hemorrhage, can also influence poor visual prognosis^20^. In the subretinal surgery group, the baseline BCVA was worse despite the SMH size was smaller than the other groups. It suggests that more patients with thick and fovea-involving SMH could have been included in the subretinal surgery group. In addition, the mean number of intravitreal anti-VEGF injections before the occurrence of SMH was lower in the anti-VEGF monotherapy group (0.19) than the other groups (Table 1, observation = 0.29, non-surgical gas tamponade = 0.48, and subretinal surgery = 2.70). This implies that the patients in the anti-VEGF monotherapy group might have had a shorter duration of nAMD and their photoreceptors in the macula might have been less damaged than the other groups. This could have resulted in more rapid visual recovery in the anti-VEGF monotherapy group than the other groups.

We performed a multicenter study including three centers with the largest number of subjects to date. However, this study has several limitations. First, the baseline variables were unbalanced among the groups due to the retrospective nature of this study. Due to the low incidence and variable spectrum of SMH following nAMD, there were limits to consider prospective study design with multiple therapeutic options. Even the prospective studies mentioned above (NCT04663750, NCT01835067, and NCT02557451) have not reported results yet, and thus, the retrospective study design is the only current option that we can find clues about the optimal treatment methods for SMH from nAMD. To overcome the potential bias inherent to the retrospective study design, we recruited a large number of consecutive patients with different treatment strategies and adjusted for the effects of multiple covariates using multivariate analysis, ANCOVA. In addition, subgroup analysis was also performed to examine whether the treatment differences depended on the baseline variables. Second, analyses using central fovea thickness that affected the choice of treatment strategy could not be performed because of measurement failure in many cases^10^. Moreover, stratification according to time-to-treatment and additional recruitment of eyes that have a wider range of time-to-treatment is needed to overcome the possibility of selection bias. The distinction between typical nAMD and PCV could not be made because the baseline SMH and subretinal scarring during follow-up inhibited angiographic differentiation in many cases. Despite these limitations, this study has the strength that it included the largest number of patients up to now from multiple centers to compare the treatment efficacy of four different strategies to treat the massive SMH from nAMD.

In conclusion, there was no difference in 1-year visual outcome among observation, anti-VEGF monotherapy, non-surgical gas tamponade, and subretinal surgery for SMH following nAMD. Anti-VEGF injection was the most effective for short-term visual improvement, especially for patients with intermediate baseline BCVA and small sized SMH.

## Supplementary Information

Supplementary Table S1.

## Data Availability

The datasets used and analyzed to support the findings of current study are available from the corresponding authors upon reasonable request.

## Abbreviations

AMD: Age-related macular degeneration
VEGF: Vascular endothelial growth factor
SRH: Subretinal hemorrhage
OCT: Optical coherence tomography
