# Supplementary material for: Comparison of treatment methods for submacular hemorrhage in neovascular age-related macular degeneration: conservative versus active surgical strategy
Source: Sci Rep. 2022 Sep 1;12:14875. doi: 10.1038/s41598-022-18619-5 (PMC9436992; doi:10.1038/s41598-022-18619-5)
Supplement: Supplementary file 1 — Supplementary Table S1. [file 41598_2022_18619_MOESM1_ESM.pdf]

Supplementary Table 1. Comparison of post-treatment visual acuities among four treatment strategies for submacular hemorrhage in neovascular age-related macular degeneration

	Observation (21)	Anti-VEGF monotherapy (161)	Non-surgical gas tamponade (31)	Subretinal surgery (23)	*P* value*
All patients, No.	21	161	31	23	
Baseline BCVA, mean (SD)	1.57 (0.74)	1.09 (0.70)	1.31 (0.83)	1.62 (0.77)	-
Post-treatment BCVA after 3 months, mean (SD)	1.67 (0.79)	0.86 (0.75)	1.41 (0.95)	1.47 (0.89)	**<.001**
Post-treatment BCVA after 6 months, mean (SD)	1.52 (0.90)	0.89 (0.80)	1.41 (0.92)	1.36 (0.88)	**.015**
Post-treatment BCVA after 12 months, mean (SD)	1.39 (0.84)	0.90 (0.83)	1.35 (0.88)	1.44 (0.91)	.204
Baseline VA ≥ 2.0 LogMAR, No.	6	11	6	9	
Baseline BCVA, mean (SD)	2.42 (0.22)	2.51 (0.33)	2.65 (0.40)	2.40 (0.47)	-
Post-treatment BCVA after 3 months, mean (SD)	2.49 (0.30)	2.09 (0.71)	1.85 (0.97)	1.66 (0.76)	.579
Post-treatment BCVA after 6 months, mean (SD)	1.99 (0.41)	2.16 (0.68)	1.95 (0.99)	1.59 (0.79)	.869
Post-treatment BCVA after 12 months, mean (SD)	1.84 (0.62)	1.88 (0.70)	1.81 (1.23)	1.49 (0.72)	.957
1.0 LogMAR ≤ Baseline BCVA < 2.0 LogMAR, No.	11	76	14	11	
Baseline BCVA, mean (SD)	1.50 (0.36)	1.48 (0.40)	1.36 (0.36)	1.27 (0.31)	-
Post-treatment BCVA after 3 months, mean (SD)	1.78 (0.54)	1.08 (0.65)	1.39 (0.83)	1.35 (0.87)	**.013**
Post-treatment BCVA after 6 months, mean (SD)	1.63 (0.70)	1.06 (0.66)	1.42 (0.84)	1.16 (0.81)	**.034**
Post-treatment BCVA after 12 months, mean (SD)	1.73 (0.60)	1.06 (0.71)	1.39 (0.85)	0.89 (0.68)	.056
Baseline VA < 1.0 LogMAR, No.	4	74	11	3	
Baseline BCVA, mean (SD)	0.47 (0.27)	0.47 (0.22)	0.53 (0.27)	0.58 (0.22)	-
Post-treatment BCVA after 3 months, mean (SD)	0.60 (0.59)	0.44 (0.53)	1.21 (1.07)	1.32 (1.49)	**.033**
Post-treatment BCVA after 6 months, mean (SD)	0.47 (0.47)	0.55 (0.72)	1.11 (0.94)	1.42 (1.46)	.188
Post-treatment BCVA after 12 months, mean (SD)	0.28 (0.37)	0.63 (0.83)	1.03 (0.69)	2.47 (0.92)	.065
Size of SMH <= 4 DD, No.	0	60	14	13	
Baseline BCVA, mean (SD)	-	0.85 (0.59)	0.96 (0.55)	1.42 (0.89)	-
Post-treatment BCVA after 3 months, mean (SD)	-	0.56 (0.56)	1.44 (1.02)	1.26 (0.96)	**.007**
Post-treatment BCVA after 6 months, mean (SD)	-	0.67 (0.72)	1.45 (0.97)	1.14 (0.85)	**.037**
Post-treatment BCVA after 12 months, mean (SD)	-	0.65 (0.74)	1.20 (1.01)	1.70 (1.07)	.097
Size of SMH > 4 DD, No.	21	101	17	10	
Baseline BCVA, mean (SD)	1.57 (0.74)	1.23 (0.73)	1.61 (0.92)	1.78 (0.65)	-
Post-treatment BCVA after 3 months, mean (SD)	1.67 (0.79)	1.04 (0.80)	1.39 (0.91)	1.63 (0.83)	.059
Post-treatment BCVA after 6 months, mean (SD)	1.52 (0.90)	1.03 (0.82)	1.39 (0.91)	1.53 (0.89)	.142
Post-treatment BCVA after 12 months, mean (SD)	1.39 (0.84)	1.04 (0.84)	1.43 (0.83)	1.27 (0.80)	.322

Abbreviations: VEGF, vascular endothelial growth factor; t-PA, tissue plasminogen activator; LogMAR, logarithm of the minimum angle of resolution; BCVA, best-corrected visual acuity; SD, standard deviation; SMH, submacular hemorrage; DD, disc diameter

*Ranked analysis of covariance with four covariates, age, baseline BCVA, size of SMH, and the number of intravitreal anti-VEGF before SMH
